# Supplementary material for: Global, regional, and national burden of hypertensive intracerebral hemorrhage, 1990 to 2021 and projections to 2050: Results from the Global Burden of Disease Study 2021
Source: PLoS One. 2025 Nov 18;20(11):e0336563. doi: 10.1371/journal.pone.0336563 (PMC12626279; doi:10.1371/journal.pone.0336563)
Supplement: Attachment — Submitted filename: Response to Reviewers.docx [file pone.0336563.s002.docx]

**Response to Reviewers**

**Manuscript ID number:**

PONE-D-25-19666

**Title of paper:**

Global, regional, and national burden of hypertensive intracerebral hemorrhage, 1990 to 2021 and projections to 2050: Results from the Global Burden of Disease Study 2021

Dear Editor and Reviewers,

We are very grateful for the opportunity to revise the manuscript entitled “Global, regional, and national burden of hypertensive cerebral hemorrhage from 1990 to 2021 and projections to 2050: results from the Global Burden of Disease Study 2021” and thank the reviewers for their insightful comments. These comments were invaluable and very helpful in revising and improving the paper, as well as providing important guidance for our research. In the submitted manuscript “Revised Manuscript with Track Changes”, we have kept all the traces of changes and made detailed corrections to the comments of the journal, reviewer 1, and reviewer 2, which are prominently highlighted in blue, yellow, and green, in that order. Below, we have provided a detailed response to each of the reviewers' comments and listed the number of lines of changes (for “Revised Manuscript with Track Changes” manuscripts) for easy reference. In addition, we have completely revised the entire manuscript. In this response letter, the reviewers' comments are italicized, and our corresponding changes and additions to the manuscript are highlighted in red. We have made every effort to make all revisions clear, and we hope that the revised manuscript will meet the requirements for publication.

In addition, there is a question for the editors to note that "the study was supported by the National Natural Science Foundation of China (82274458)" has been added to the manuscript funding section, and this project has also been added to the system. We apologize for any inconvenience caused, and please contact us if there is anything unreasonable.

**Journal Requirements:**

*When submitting your revision, we need you to address these additional requirements.*

*1. Please ensure that your manuscript meets PLOS ONE's style requirements, including those for file naming. The PLOS ONE style templates can be found at*

*https://journals.plos.org/plosone/s/file?id=wjVg/PLOSOne_formatting_sample_main_body.pdf and*

*<https://journals.plos.org/plosone/s/file?id=ba62/PLOSOne_formatting_sample_title_authors_affiliations.pdf>*

**We have checked and revised the style requirements and file naming requirements of the manuscript according to the PLOS ONE style template provided by you to ensure that the revised manuscript meets the requirements of your journal.**

*2. Please include your full ethics statement in the ‘Methods’ section of your manuscript file. In your statement, please include the full name of the IRB or ethics committee who approved or waived your study, as well as whether or not you obtained informed written or verbal consent. If consent was waived for your study, please include this information in your statement as well.*

**We have included a full ethical statement in the “Methods” section of the manuscript document. The full name of the ethics committee that approved or exempted the study has also been indicated and the exemption of the study from obtaining informed consent has been explained. In the “Revised Manuscript with Track Changes”, lines 121-126.**

*3. Please ensure that you refer to Figure 9 to 13 in your text as, if accepted, production will need this reference to link the reader to the figure.*

**Figures 9 to 13, referred to in the text, have been redeployed and elaborated in order to better connect the reader to the figures. In the “Revised Manuscript with Track Changes”, lines 339-360.**

*4. Please upload a copy of Supporting Information Figure/Table/etc. “Supporting information” which you refer to in your text on page 27.*

**We are going to elaborate on this issue, due to our misinterpretation, the two tables with 13 images covered in the main text were written into the supplementary material, i.e., the “Supporting Information” on page 27 in the first version of the submitted manuscript is the same as the charts and graphs in the main text and no new charts/tables, etc., of supporting information have been added, so the supplementary material This section is not available to us and this section should be deleted. We apologize for any confusion caused to you. In the “Revised Manuscript with Track Changes”, lines 809-832.**

**Response to reviewer #1:**

Dear Reviewer #1,

Thank you for your constructive comments on my manuscript. I appreciate your time and effort in reviewing my paper and providing valuable suggestions. I have revised manuscript according to your feedback and addressed each point in detail below.

Thank you again for reviewing the manuscript, and I hope my revisions meet your requirements.

***Reviewer #1:*** *The manuscript analyzes the global, regional, and national burden of hypertensive intracerebral hemorrhage (HICH) from 1990 to 2021 using data from the Global Burden of Disease (GBD) 2021 study. It focuses on age-standardized death rates (ASDR) and disability-adjusted life years (DALYs), examining trends across countries, regions, age groups, sexes, and sociodemographic index (SDI) levels. The study reports a significant decline in global ASDR (31.418%) and DALYs (32.163%) from 1990 to 2021, with projections to 2050 using a Bayesian age-period-cohort (BAPC) model indicating continued declines. The authors highlight persistent disparities in less developed regions and call for targeted prevention and treatment strategies to address these gaps.*

***Areas of improvement:***

***1. Limited Discussion of HICH-Specific Risk Factors:***

*While the manuscript identifies hypertension as the primary risk factor for HICH, it does not sufficiently explore other contributing factors (e.g., smoking, alcohol use, obesity) or their interactions with hypertension. The discussion of risk factors is brief and relies heavily on general statements.*

***Recommendation:*** *Expand the discussion to include a detailed analysis of HICH-specific risk factors, supported by GBD data on attributable fractions or relevant literature. Discuss how these factors vary by region, sex, or SDI.*

**Your comments and suggestions are much appreciated and we have referred to the relevant literature to further analyze the specific risk factors for HICH and to further discuss how these factors differ by region, gender, or SDI. In the “Revised Manuscript with Track Changes”, lines 85-100.The additions are specified below:**

**In addition, factors such as hypertension, diet, and the environment can also have a significant impact on HICH risk across different regions, genders, and contexts such as the SDI[1].High systolic blood pressure is the most important factor for high ASDRs and age-standardized DALYs rates for ICH in 2021, followed by particulate matter pollution, cigarette smoking, indoor air pollution from solid fuels, and a high sodium diet, and there were gender differences in these risks, with the main factors influencing the risk of death for males being smoking and particulate matter pollution of the atmosphere, and the main factors influencing the risk of death for females being particulate matter pollution and indoor air pollution from solid fuels[2]. Among the different regions, Central and Southeast Asia have a higher prevalence of hypertension, which is coupled with unhealthy lifestyles such as high-salt diets and smoking, which further adds to the burden of ICH[3]. In low SDI areas, particulate matter pollution and smoking account for only 8.1% and 5.9% of all deaths from ICH, respectively, and household air pollution from solid fuels can account for 40.2% of all deaths. However, in areas with high SDI levels, deaths due to particulate matter pollution, smoking, and household air pollution are lower[2]**

***2. Inadequate Exploration of Data Limitations:***

*The manuscript acknowledges variability in data quality, particularly in low- and middle-income countries, but does not thoroughly discuss the implications of these limitations. For example, it does not address potential biases in HICH diagnosis, underreporting, or differences in healthcare access that may affect GBD estimates.*

***Recommendation:*** *Dedicate a paragraph in the discussion to explore data limitations, including diagnostic accuracy, data completeness, and the ecological study design’s inability to infer causality. Discuss how these limitations may impact the findings and projections.*

**Thank you very much for your comments and suggestions, we have included a paragraph exploring the limitations of the data including diagnostic accuracy, data completeness, and the inability of ecological study designs to infer causality and discussing how these limitations may affect study results and predictions. In the “Revised Manuscript with Track Changes”, lines 526-561.The additions are specified below:**

**Strengths and limitations**

**The strength of this study lies in its systematic analysis of spatial and temporal trends in ASDRs and age-standardized DALYs rates for HICH by country, region, age, sex, and level of SDI using data from the GBD study from 1990 to 2021. We also projected the global burden of ASDR, age-standardized DALYs rates for HICH to 2050. To the best of our knowledge, this GBD-based study represents one of the more comprehensive efforts to date to analyze the global burden of HICH in terms of mortality rates, DALYs, and projected future trends; however, as described in previous studies[4-7], there are some limitations to the present study: Firstly, there may be challenges in diagnosing and reporting hypertensive cerebral hemorrhagic disease, i.e., variations in diagnostic criteria, reporting standards, and data collection methods in different countries and regions can affect the accuracy of results. Second, when GBD collects and organizes regional data, there may be incomplete data for some regions. For example, some studies may not provide regional data, while others may provide data that are inconsistent with GBD's regional delineation. In addition, the GBD database incorporates a relatively wide range of data, and the quality and availability of data vary significantly across countries and regions, especially in low- and middle-income countries that mostly lack robust data registries. As a result, the regional burden of HICH may not be accurately estimated, thereby affecting the effectiveness of targeted interventions and resource allocation. Third, GBD analyses rely on statistical models and assumptions to make inferences, which to some extent ignores real-world complexity factors and may not better reflect the lived experiences of people with HICH in different cultural contexts. Fourth, no risk factors for HICH were identified in the GBD database. HICH is a multifactorial disease, and its pathogenesis is related to genetics, environment, and lifestyle in addition to hypertension as a major factor. This study may not provide a comprehensive understanding of the risk factors for HICH, which to some extent hinders the development of effective prevention and control strategies. Finally, the impact of time should be considered. This analysis spanned a period of 30 years, and projections of the future burden of HICH were mostly based on current trends and patterns, during which major developments in medical technology, treatment protocols, and public health policies may have had an impact on the burden of HICH, which to a certain extent ignored the possible intervening factors that may have been relevant in the passage of time. Future research should focus on the impact of health policies, resource allocation, etc., and utilize multidisciplinary approaches and technologies to enhance the prevention, treatment, and global health outcomes of HICH.**

***3. Overreliance on Descriptive Statistics:***

*The results section is heavily descriptive, presenting extensive numerical data (e.g., ASDR, DALYs, EAPC) without sufficient synthesis or interpretation. This makes the section dense and challenging to follow for readers unfamiliar with GBD studies.*

***Recommendation:*** *Streamline the results section by focusing on key trends and using tables/figures to summarize data. Provide more interpretive commentary to highlight the significance of findings (e.g., why certain regions have higher burdens).*

**Thank you very much for your comments and suggestions, we have streamlined the results section somewhat by focusing on the discussion of major trends and using tables/graphs to summarize the data, as well as providing more explanatory comments in the discussion section to emphasize the importance of the findings. In the “Revised Manuscript with Track Changes”, lines 121-364.Due to the large and mostly detailed issues involved, they are not listed separately here and have been labeled in the text.**

***4. Insufficient Detail on BAPC Model:***

*The manuscript briefly describes the BAPC model but does not explain its assumptions, limitations, or how it handles uncertainty in projections. Readers unfamiliar with this method may struggle to assess its validity.*

***Recommendation:*** *Expand the methods section to include a detailed explanation of the BAPC model, including its mathematical framework, assumptions (e.g., stationarity of trends), and how it accounts for future changes in risk factors or healthcare access. Discuss potential limitations of long-term projections.*

**Thank you very much for your comments and suggestions, and we have expanded the Methods section to explain the BAPC model in detail, including its mathematical framework, assumptions, and how it takes into account future changes in risk factors or healthcare delivery, and to discuss potential limitations of long-term projections. In the “Revised Manuscript with Track Changes”, lines 161-177.The additions are specified below:**

**A Bayesian age-period-cohort (BAPC) model was employed to predict the ASDR, the DALYs, and the age-standardized DALYs rate for HICH from 2025 to 2050. BAPC modeling is a methodology used in epidemiology and biostatistics to analyze the relationship between incidence rates and time. It uses sample data and a priori information to obtain unique parameter estimates[8], allows for the inclusion of known risk factors as covariates in the model, and also simulates the impact of future changes in healthcare by setting up different scenarios. Based on the assumption that the effects of age, period, and cohort are similar in time, the Bayesian inference in the BAPC model utilizes second-order stochastic bias to smooth the three aforementioned prior values and predict the posterior rate[2].BAPC employs the Integrated Nested Laplace Approximation (INLA) to approximate the marginal posterior distributions, thereby avoiding the mixing and convergence problems associated with the Markov Chain Monte Carlo method and the traditional Bayesian approach , which has been widely used to analyze trends in chronic diseases and predict future disease burden[9]. Its long-term predictions are prone to deviate from the assumed conditions, and data quality and availability can suffer from the difficulty of incorporating unpredictable events.**

***5. Generic Policy Recommendations:***

*The policy recommendations (e.g., increasing screening, improving medical facilities) are broad and lack specificity regarding implementation, cost-effectiveness, or feasibility in low-resource settings. The manuscript does not address potential barriers, such as political or cultural resistance.*

***Recommendation:*** *Provide more concrete policy recommendations, such as integrating HICH screening into existing hypertension programs, leveraging telemedicine for rural areas, or adopting cost-effective interventions like community health worker training. Discuss implementation challenges and potential solutions.*

**Your comments and suggestions are greatly appreciated, and we have addressed implementation challenges and potential solutions in the discussion section, as well as provided more specific policy recommendations, such as incorporating HICH screening into existing hypertension programs, utilizing telemedicine to serve rural areas, or cost-effective interventions such as community health worker training. In the “Revised Manuscript with Track Changes”, lines 461-464,562-590.The additions are specified below:**

**Strengthening medical infrastructure and public health services, such as integrating screening for HICH into existing hypertension programs, building regional telemedicine networks to serve rural areas, and training community health workers, are critical to reducing the burden of HICH in these regions.**

**Future directions**

**Recommendations for future research and policy are essential to maintain and enhance HICH disease control. Further research should focus on understanding the underlying causes of ASDR and age-standardized DALYs rate anomalies in HICH in regions such as sub-Saharan Africa and on exploring effective interventions tailored to the local conditions, which may include examining socio-cultural, economic, and environmental factors influencing HICH, such as exploring the morbidity and mortality of** **HICH under the influence of different local cultures and religious beliefs; investigating the distribution, treatment, and prognosis of HICH in different areas with different local economic development statuses. Policy initiatives should prioritize the strengthening of healthcare infrastructure and accessibility in low- and middle- SDI regions, such as local governments formulating specific policies and plans for the prevention and control of HICH and incorporating them into the priorities of national public health strategies; establish bilateral or multilateral cooperation with relevant countries to share prevention and control experience and technology; expand health insurance coverage for low-income groups and increase the reimbursement rate of medical expenses related to HICH so as to alleviate the burden on patients and reduce their financial burden; train professional health workers to provide guidance and education so as to increase the rate of knowledge and management of the disease; set up a telemedicine platform to realize real-time connectivity between higher-level hospitals and primary healthcare institutions, and at the same time, strengthen the construction of medical informationization, and set up a database of patients' health records so as to facilitate the long-term tracking and management of patients with hypertension; and promote healthy living through a variety of channels, such as community activities, media publicity and school education. Implementing socio-economic development programs to improve living conditions, nutrition and education, such as strengthening air pollution control, providing safe and hygienic drinking water, etc., building more public fitness venues and facilities, encouraging residents to actively participate in physical exercise, and fostering a healthy social atmosphere.**

***6. Inconsistent Temporal References:***

*The manuscript inconsistently refers to the study period (e.g., “2019” in Tables 1 and 2 instead of 2021, “32 years” vs. “1990–2021”). This creates confusion about the data’s temporal scope.*

***Recommendation:*** *Standardize all temporal references to 1990–2021 and correct errors in tables (e.g., replace “2019” with “2021”). Ensure consistency in describing the study period throughout the manuscript.*

**Thank you very much for your comments and suggestions. we have standardized all time citations to 1990-2021 and corrected the error in the table by replacing “2019” with “2021” to ensure that the time period of the study is described consistently in the manuscript. In the “Revised Manuscript with Track Changes”, lines 51,211.**

***7. Underutilized Supplementary Materials:***

*The manuscript references 13 supplementary figures but does not adequately integrate them into the main text. For example, S9–S13 figures are cited but not summarized, limiting their utility for readers.*

***Recommendation:*** *Provide brief summaries of key supplementary findings in the main text or include a dedicated section describing the supplementary materials. Ensure all figures are clearly labeled and cross-referenced.*

**Thank you very much for your comments and suggestions. We are going to elaborate on this issue, due to our misinterpretation, the two tables with 13 images covered in the main text were written into the supplementary material, i.e., the “Supporting Information” on page 27 in the first version of the submitted manuscript is the same as the charts and graphs in the main text and no new charts/tables, etc., of supporting information have been added, so the supplementary material This section is not available to us and this section should be deleted. We apologize for any confusion caused to you. In the “Revised Manuscript with Track Changes”, lines 809-832.**

***8. Minor Editorial Issues:***

*The manuscript contains minor typographical errors (e.g., “supplemalestary” in some sections, inconsistent spacing in references) and awkward phrasing (e.g., “more severe sites” for HICH in males). These detract from the manuscript’s professionalism.*

***Recommendation:*** *Conduct a thorough proofreading to correct typos, standardize terminology, and improve sentence clarity. Ensure references adhere to PLOS ONE’s formatting guidelines.*

**Thank you very much for your comments and suggestions. We have thoroughly proofread the manuscript to correct typos, standardize terminology, and improve sentence clarity to ensure that references comply with PLOS ONE's formatting guidelines.**

**Response to reviewer #2:**

Dear Reviewer #2,

Thank you very much for your time and effort in providing valuable comments on my manuscript. I have revised my manuscript based on your feedback and have elaborated on each point below.

Thank you again for reviewing the manuscript, and I hope my revisions meet your requirements.

***Reviewer #2:*** *I have reviewed the manuscript analyzing trends in hemorrhagic intracerebral hemorrhage (HICH) using Global Burden of Disease data. I believe that this work provides valuable epidemiological insights, but I think that several minor revisions are needed before publication.*

***Introduction:***

*The progression from global ICH burden to HICH specifics and then to GBD data trends is somewhat abrupt. I would fix this flow, although it is still clear in a way, but it would be further enhanced with more logical transitions.*

**Thank you very much for your comments and suggestions. We have revised the introductory section from the global ICH burden to HICH specifics to GBD data trends to make the transition logical and clear. In the “Revised Manuscript with Track Changes”, lines 61-85. The additions are specified below:**

**Intracerebral hemorrhage (ICH) is the most devastating form of stroke[10], contributing significantly to global morbidity and mortality[11], and imposing a substantial burden on healthcare systems worldwide[12].Hypertensive intracerebral hemorrhage (HICH) is the predominant subtype of ICH and is strongly associated with hypertension，accounts for approximately 70% of all ICH cases[13].** **Advances in detection methods, improved treatments, and heightened awareness have influenced the morbidity and mortality rates of ICH[14].A study based on computed tomography found that noncontrast CT has a sensitivity of up to 95% in the detection of ICH and has been widely used[15]. Studies on the treatment of ICH have found that blood pressure control trials reduce ICH mortality by 10-15%[16]and antiplatelet/anticoagulants improve the prognosis of ICH by 30-40%[17]. Public health campaigns focusing on hypertension control increased regular blood pressure checks by 20-30% in some regions and reduced smoking rates by 10-15% in certain communities, thereby lowering ICH risk[18].**

*I think the introduction is good, but in my opinion, it lacks a clear statement of the knowledge gap or why analysing HICH trends specifically by geography, SDI, age, and gender is important. This would enhance the introduction and emphasise the importance and strength of the study. I would highly suggest that you fix this.*

**Thank you very much for your comments and suggestions. We have revised the introductory section to include the importance of specific analyses of HICH trends by geography, SDI, age, and gender, thereby enhancing the introduction and emphasizing the importance and strengths of the study. In the “Revised Manuscript with Track Changes”, lines 85-107. The additions are specified below:**

**In addition, factors such as hypertension, diet, and the environment can also have a significant impact on HICH risk across different regions, genders, and contexts such as the SDI[1].High systolic blood pressure is the most important factor for high ASDRs and age-standardized DALYs rates for ICH in 2021, followed by particulate matter pollution, cigarette smoking, indoor air pollution from solid fuels, and a high sodium diet, and there were gender differences in these risks, with the main factors influencing the risk of death for males being smoking and particulate matter pollution of the atmosphere, and the main factors influencing the risk of death for females being particulate matter pollution and indoor air pollution from solid fuels[2]. Among the different regions, Central and Southeast Asia have a higher prevalence of hypertension, which is coupled with unhealthy lifestyles such as high-salt diets and smoking, which further adds to the burden of ICH[3]. In low SDI areas, particulate matter pollution and smoking account for only 8.1% and 5.9% of all deaths from ICH, respectively, and household air pollution from solid fuels can account for 40.2% of all deaths. However, in areas with high SDI levels, deaths due to particulate matter pollution, smoking, and household air pollution are lower[2].Thus, HICH has emerged as a major public health problem as a leading cause of death and long-term disability among adults worldwide, especially in low- and middle-income countries[19-21]. In addition, its main risk factor, high systolic blood pressure, is closely related to factors such as smoking, diet, and the environment and shows great variations depending on the country, region, gender, and SDI level, among others. Therefore, a detailed analysis of the spatial and temporal trends, gender and age differences in the epidemiology of HICH is needed to guide prevention and treatment.**

*My last suggestion for this section is the referencing of literature. Some references (e.g. [5], [6]) are cited vaguely, and it is unclear what specific data or findings they refer to. If you further emphasise using numbers or statements from the studies you have referenced, you can make the reader more intrigued to read your research, since you have built a strong introduction.*

**Thank you very much for your comments and suggestions. We have expanded the cited references by using specific data or conclusions. In the “Revised Manuscript with Track Changes”, lines76-85. The additions are specified below:**

**Studies on the treatment of ICH have found that blood pressure control trials reduce ICH mortality by 10-15%[16]and antiplatelet/anticoagulants improve the prognosis of ICH by 30-40%[17]. Public health campaigns focusing on hypertension control increased regular blood pressure checks by 20-30% in some regions and reduced smoking rates by 10-15% in certain communities, thereby lowering ICH risk[18]. In addition, a stroke study based on the GBD database from 1990 to 2019 found a 17.0% decrease in incidence, a 60% decrease in prevalence, a 36.0% decrease in mortality and disability-adjusted life years (DALYs) for ICH compared with ischemic stroke[22]. However, these improvements are not uniform, and disparities remain across countries, regions, sociodemographic index (SDI) levels, gender, etc.**

***Methods:***

*Generally, this section is well written and explained. There are some areas I would like to draw attention to, and I am saying this because I am a first-time reader, so my comments might be due to my confusion rather than my knowledge.*

*The phrase "queried raw data" is vague and needs clarification. I asked myself, "Did the authors extract incidence, mortality, DALYs, or other variables from GBD 2021? Was it done through the GHDx tool or GBD Results Tool?" I would suggest elaborating on this section further to enhance the strength of the methods used to conduct this strong study.*

**Thank you very much for your comments and suggestions. In the “Data Sources” section, we have further elaborated and explained the “Raw Data Queried” and also further elaborated on the relationship between the variables of mortality, DALYs, etc. and 2021GBD to enhance the strength of the research methodology. In the “Revised Manuscript with Track Changes”, lines76-85. The additions are specified below:**

**This study is part of the GBD 2021 update, which estimates epidemiological information for 204 countries and territories and provides comparative analyses of 369 diseases and injuries[23]. We obtained data on the burden of HICH from 1990 to 2021 from the Global Health Data Exchange query tool (GHDx) (http://ghdx.healthdata.org/gbd-results-tool).The methods of data acquisition, processing, and analysis for the GBD 2021 study have been detailed in prior publications documented[24, 25]. Using the GBD 2021 dataset, we extracted the mortality rates, DALYs, and their corresponding age-standardized rates (ASRs) for HICH for the period 1990 to 2021.**

*While the SDI breakdown is correct, I am confused about how the SDI is assigned to each country. The method used to assign countries to each SDI category should be cited or briefly explained (e.g. based on the GBD-defined quintiles for each year). This would be enhanced if you added a statement with a reference stating that each SDI is assigned to the countries using WHO categorisations. Explaining or emphasising standards here is very important because of the study aim; otherwise, it will be very vague and subject to critiques.*

**Thank you very much for your comments and suggestions. We have provided a description of the methodology used to assign countries to each of the SDI categories and added relevant references to illustrate the sources of the categorization methodology. In the “Revised Manuscript with Track Changes”, lines147-155. The additions are specified below:**

**The SDI data used in this study came from the Institute for Health Metrics and Evaluation (IHME)[26]. It categorized all GBD 2021 locations into SDI quintiles based on the calculated SDI values for 2021. The low SDI, low-medium SDI, medium SDI, high-medium SDI, and high SDI quintiles were generated based on the quintiles defined by the GBD for each year. Specifically, the SDI categorizes areas into five classes based on the following ranges: low SDI (0-0.454743), low-medium SDI (0.454743-0.607679), medium SDI (0.607679-0.689504), high-medium SDI (0.689504-0.805129), and high SDI (0.805129-1).**

*Also, the BAPC model is mentioned, but I am confused about how this model was used, designed, or validated. This section is very concise and lacks clarity in some places. Again, I believe that emphasising this section by detailing the methodological approach is essential.*

**Thank you very much for your comments and suggestions. We have described and illustrated the computation, use, design and validation of the BAPC model. In the “Revised Manuscript with Track Changes”, lines161-177. The additions are specified below:**

**A Bayesian age-period-cohort (BAPC) model was employed to predict the ASDR, the DALYs, and the age-standardized DALYs rate for HICH from 2025 to 2050. BAPC modeling is a methodology used in epidemiology and biostatistics to analyze the relationship between incidence rates and time. It uses sample data and a priori information to obtain unique parameter estimates[8], allows for the inclusion of known risk factors as covariates in the model, and also simulates the impact of future changes in healthcare by setting up different scenarios. Based on the assumption that the effects of age, period, and cohort are similar in time, the Bayesian inference in the BAPC model utilizes second-order stochastic bias to smooth the three aforementioned prior values and predict the posterior rate[2].BAPC employs the Integrated Nested Laplace Approximation (INLA) to approximate the marginal posterior distributions, thereby avoiding the mixing and convergence problems associated with the Markov Chain Monte Carlo method and the traditional Bayesian approach , which has been widely used to analyze trends in chronic diseases and predict future disease burden[9]. Its long-term predictions are prone to deviate from the assumed conditions, and data quality and availability can suffer from the difficulty of incorporating unpredictable events.**

*Instead of "a p-value < for 0.05…", I would say "A p-value < 0.05 was considered statistically significant." How was the EAPC calculated? This is also not clear.*

**Thank you very much for your comments and suggestions. We have changed “p-value less than 0.05 ......” to “p-value less than 0.05 are considered statistically significant”.** **In the “Revised Manuscript with Track Changes”, lines 190.**

**We also further elaborate on the calculation of EAPC. In the “Revised Manuscript with Track Changes”, lines 182-190. The additions are specified below:**

**The EAPC is a commonly used metric in epidemiological studies to assess trends in age-standardized ratios (ASRs) of diseases over time. The coefficient** $\boldsymbol{\beta}$ **is derived from the natural logarithm of the ASRs, where** $\boldsymbol{y}$ **represents** $\boldsymbol{In}$**(ASR) and** $\boldsymbol{x}$ **represents calendar years. The EAPC and its 95% CI were determined by the following linear regression model:** $\boldsymbol{y=\alpha+\beta x+\varepsilon EAPC=100*(}\exp\left( \boldsymbol{\beta} \right)\boldsymbol{-1)}$**[27].** **When the lower limit of the 95% CI exceeds 0, it indicates an upward trend; when the upper limit is below 0, it indicates a downward trend. If the 95% CI contains 0, it indicates no statistically significant change in the trend pattern. In trend analysis, *P*<0.05 is considered statistically significant.**

***Results:***

*The results state that trends are "significant”, but there is no mention of p-values or interpretation of CIs (although the CIs do not cross 0, indicating statistical significance). I would suggest adding the P value here.*

**Thank you very much for your comments and suggestions. We have already explained the CIs. In the “Revised Manuscript with Track Changes”, lines 187-190. The additions are specified below:**

**When the lower limit of the 95% CI exceeds 0, it indicates an upward trend; when the upper limit is below 0, it indicates a downward trend. If the 95% CI contains 0, it indicates no statistically significant change in the trend pattern.**

**We added p-values to some of the results after reviewing the relevant literature. In the “Revised Manuscript with Track Changes”, lines 295-302. The additions are specified below:**

**We also explored the relationship between ASDRs, age-standardized DALYs rates, and SDI. The results, shown in Fig 7 and 8, indicate that there is a significant negative correlation between SDI and ASDR (*ρ*=-0.687,*P*<0.05) and age-standardized DALYs rate (*ρ*=-0.700,*P*<0.05) at the geographic level (Fig 7), and that there is a significant negative correlation between SDI and ASDR (*ρ*=-0.762,*P*<0.05), and age-standardized DALYs rate (*ρ*=-0.769,*P*<0.05) at the country level (Fig 8), suggesting that geographic areas or countries with higher SDIs may have lower ASDRs and age-standardized DALYs rates for HICH.**

***Discussion and Conclusion:***

*"The results of this study are generally consistent with those of previous studies showing an overall decreasing trend in the global burden of cerebral haemorrhage; however, the authors have not provided the actual numbers, which weakens the comparison and decreases the study strength in adding value to the current literature. Other sentences with the same structure in this section also lack numbers or comparison details.*

**Thank you very much for your comments and suggestions. We have provided actual numbers in this section and have provided actual numbers or comparative details in other sentences with the same structure in this section. In the “Revised Manuscript with Track Changes”, lines 400-525. As this section is more dispersed and labeled in the article, it will not be listed separately.**

*I also noticed the discussion is mostly descriptive and lacks in-depth causal interpretation.*

**Thank you very much for your comments and suggestions. We have revised the discussion section by reducing the use of descriptive statements and adding in-depth causal explanations. In the “Revised Manuscript with Track Changes”, lines 366-525. As this section is more dispersed and labeled in the article, it will not be listed separately.**

*I noticed that the limitations and strengths section is missing. You may have merged it with the text, but I would highly recommend that you do a separate section.*

**Thank you very much for your comments and suggestions. We have separated out the “Limitations and Strengths” section. In the “Revised Manuscript with Track Changes”, lines 526-561.**

**Strengths and limitations**

**The strength of this study lies in its systematic analysis of spatial and temporal trends in ASDRs and age-standardized DALYs rates for HICH by country, region, age, sex, and level of SDI using data from the GBD study from 1990 to 2021. We also projected the global burden of ASDR, age-standardized DALYs rates for HICH to 2050. To the best of our knowledge, this GBD-based study represents one of the more comprehensive efforts to date to analyze the global burden of HICH in terms of mortality rates, DALYs, and projected future trends; however, as described in previous studies[4-7], there are some limitations to the present study: Firstly, there may be challenges in diagnosing and reporting hypertensive cerebral hemorrhagic disease, i.e., variations in diagnostic criteria, reporting standards, and data collection methods in different countries and regions can affect the accuracy of results. Second, when GBD collects and organizes regional data, there may be incomplete data for some regions. For example, some studies may not provide regional data, while others may provide data that are inconsistent with GBD's regional delineation. In addition, the GBD database incorporates a relatively wide range of data, and the quality and availability of data vary significantly across countries and regions, especially in low- and middle-income countries that mostly lack robust data registries. As a result, the regional burden of HICH may not be accurately estimated, thereby affecting the effectiveness of targeted interventions and resource allocation. Third, GBD analyses rely on statistical models and assumptions to make inferences, which to some extent ignores real-world complexity factors and may not better reflect the lived experiences of people with HICH in different cultural contexts. Fourth, no risk factors for HICH were identified in the GBD database. HICH is a multifactorial disease, and its pathogenesis is related to genetics, environment, and lifestyle in addition to hypertension as a major factor. This study may not provide a comprehensive understanding of the risk factors for HICH, which to some extent hinders the development of effective prevention and control strategies. Finally, the impact of time should be considered. This analysis spanned a period of 30 years, and projections of the future burden of HICH were mostly based on current trends and patterns, during which major developments in medical technology, treatment protocols, and public health policies may have had an impact on the burden of HICH, which to a certain extent ignored the possible intervening factors that may have been relevant in the passage of time. Future research should focus on the impact of health policies, resource allocation, etc., and utilize multidisciplinary approaches and technologies to enhance the prevention, treatment, and global health outcomes of HICH.**

*I believe this manuscript addresses an important topic with significant public health implications. A comprehensive approach examining variations by geography, SDI, age, and sex offers valuable perspectives for targeted interventions. With the suggested revisions to enhance methodological clarity, narrative flow, and interpretive depth, I believe this work will make a significant contribution to understanding global HICH patterns and trends.*

**Thank you again for your diligent review and suggestions, which we have taken into account to improve the clarity of the methodology, the fluency of the narrative, and the depth of the explanations, with a view to contributing to the understanding of global HICH patterns and trends.**

*I recommend this manuscript for publication following moderate revisions.*

Thank you for your comments on my paper. I appreciate your feedback and suggestions. I will revise my paper according to your comments and submit a revised version soon. I hope you will find the revised paper satisfactory and acceptable for publication. Thank you for your time and effort.

Sincerely, [Chao Zhang]

**Reference**

1.	Xu L, Wang Z, Wu W, Li M, Li Q. Global, regional, and national burden of intracerebral hemorrhage and its attributable risk factors from 1990 to 2021: results from the 2021 Global Burden of Disease Study. BMC Public Health. 2024;24(1):2426. Epub 2024/09/07. doi: 10.1186/s12889-024-19923-7. PubMed PMID: 39243077; PubMed Central PMCID: PMCPMC11378620.

2.	Song D, Xu D, Li M, Wang F, Feng M, Badr A, et al. Global, regional, and national burdens of intracerebral hemorrhage and its risk factors from 1990 to 2021. Eur J Neurol. 2025;32(1):e70031. Epub 2024/12/28. doi: 10.1111/ene.70031. PubMed PMID: 39731311; PubMed Central PMCID: PMCPMC11680743.

3.	Schutte AE, Srinivasapura Venkateshmurthy N, Mohan S, Prabhakaran D. Hypertension in Low- and Middle-Income Countries. Circ Res. 2021;128(7):808-26. Epub 2021/04/02. doi: 10.1161/circresaha.120.318729. PubMed PMID: 33793340; PubMed Central PMCID: PMCPMC8091106.

4.	Chen X, Tang H, Lin J, Zeng R. Temporal trends in the disease burden of osteoarthritis from 1990 to 2019, and projections until 2030. PLoS One. 2023;18(7):e0288561. Epub 2023/07/24. doi: 10.1371/journal.pone.0288561. PubMed PMID: 37486949; PubMed Central PMCID: PMCPMC10365297.

5.	Zhang J, Zhu Y, Wang J, Hu H, Jin Y, Mao X, et al. Global burden and epidemiological prediction of polycystic ovary syndrome from 1990 to 2019: A systematic analysis from the Global Burden of Disease Study 2019. PLoS One. 2024;19(7):e0306991. Epub 2024/07/18. doi: 10.1371/journal.pone.0306991. PubMed PMID: 39024211; PubMed Central PMCID: PMCPMC11257291.

6.	Li X, Li Y, Guo L, Chen Y, Wang G, Zhang H. Tuberculosis incidence, deaths and disability-adjusted life years in children and adolescence, 1990-2021: Results from the Global Burden of Disease Study 2021. PLoS One. 2025;20(3):e0317880. Epub 2025/03/10. doi: 10.1371/journal.pone.0317880. PubMed PMID: 40063613; PubMed Central PMCID: PMCPMC11892809.

7.	Li R, Li Y, Xiong C, Gao W. Burden of Chronic obstructive pulmonary disease in China: an analysis based on the GBD 2021 compared with the United States. PLoS One. 2025;20(4):e0321470. Epub 2025/04/17. doi: 10.1371/journal.pone.0321470. PubMed PMID: 40245085; PubMed Central PMCID: PMCPMC12005532.

8.	Fosse E, Winship C. Bounding Analyses of Age-Period-Cohort Effects. Demography. 2019;56(5):1975-2004. Epub 2019/08/30. doi: 10.1007/s13524-019-00801-6. PubMed PMID: 31463797.

9.	Chen J, Li C, Bu CLN, Wang Y, Qi M, Fu P, et al. Global burden of non-communicable diseases attributable to kidney dysfunction with projection into 2040. Chin Med J (Engl). 2024. Epub 2024/05/29. doi: 10.1097/cm9.0000000000003143. PubMed PMID: 38809055.

10.	Xu X, Zhang J, Yang K, Wang Q, Chen X, Xu B. Prognostic prediction of hypertensive intracerebral hemorrhage using CT radiomics and machine learning. Brain Behav. 2021;11(5):e02085. Epub 2021/02/25. doi: 10.1002/brb3.2085. PubMed PMID: 33624945; PubMed Central PMCID: PMCPMC8119849.

11.	Hemphill JC, Greenberg SM, Anderson CS, Becker K, Bendok BR, Cushman M, et al. Guidelines for the Management of Spontaneous Intracerebral Hemorrhage. Stroke. 2015;46(7):2032-60. doi: 10.1161/STR.0000000000000069.

12.	Al-Kawaz MN, Hanley DF, Ziai W. Advances in Therapeutic Approaches for Spontaneous Intracerebral Hemorrhage. Neurotherapeutics. 2020;17(4):1757-67. Epub 2020/07/29. doi: 10.1007/s13311-020-00902-w. PubMed PMID: 32720246; PubMed Central PMCID: PMCPMC7851203.

13.	Meretoja A, Strbian D, Putaala J, Curtze S, Haapaniemi E, Mustanoja S, et al. SMASH-U: a proposal for etiologic classification of intracerebral hemorrhage. Stroke. 2012;43(10):2592-7. Epub 2012/08/04. doi: 10.1161/strokeaha.112.661603. PubMed PMID: 22858729.

14.	Hostettler IC, Seiffge DJ, Werring DJ. Intracerebral hemorrhage: an update on diagnosis and treatment. Expert Rev Neurother. 2019;19(7):679-94. Epub 2019/06/13. doi: 10.1080/14737175.2019.1623671. PubMed PMID: 31188036.

15.	Shahrestani S, Wishart D, Han SMJ, Strickland BA, Bakhsheshian J, Mack WJ, et al. A systematic review of next-generation point-of-care stroke diagnostic technologies. Neurosurg Focus. 2021;51(1):E11. Epub 2021/07/02. doi: 10.3171/2021.4.Focus21122. PubMed PMID: 34198255.

16.	Wood H. Nature Reviews Neurology: the early years. Nat Rev Neurol. 2015;11(11):607. Epub 2015/11/05. doi: 10.1038/nrneurol.2015.204. PubMed PMID: 26530668.

17.	Steiner T, Weitz JI, Veltkamp R. Anticoagulant-Associated Intracranial Hemorrhage in the Era of Reversal Agents. Stroke. 2017;48(5):1432-7. Epub 2017/04/13. doi: 10.1161/strokeaha.116.013343. PubMed PMID: 28400486.

18.	Thrift AG, McNeil JJ, Forbes A, Donnan GA. Risk factors for cerebral hemorrhage in the era of well-controlled hypertension. Melbourne Risk Factor Study (MERFS) Group. Stroke. 1996;27(11):2020-5. Epub 1996/11/01. doi: 10.1161/01.str.27.11.2020. PubMed PMID: 8898809.

19.	Hankey GJ. Stroke. Lancet. 2017;389(10069):641-54. Epub 2016/09/18. doi: 10.1016/s0140-6736(16)30962-x. PubMed PMID: 27637676.

20.	Sheth KN. Spontaneous Intracerebral Hemorrhage. N Engl J Med. 2022;387(17):1589-96. Epub 2022/10/28. doi: 10.1056/NEJMra2201449. PubMed PMID: 36300975.

21.	Kim J, Thayabaranathan T, Donnan GA, Howard G, Howard VJ, Rothwell PM, et al. Global Stroke Statistics 2019. Int J Stroke. 2020;15(8):819-38. Epub 2020/03/10. doi: 10.1177/1747493020909545. PubMed PMID: 32146867.

22.	Global, regional, and national burden of stroke and its risk factors, 1990-2019: a systematic analysis for the Global Burden of Disease Study 2019. Lancet Neurol. 2021;20(10):795-820. Epub 2021/09/07. doi: 10.1016/s1474-4422(21)00252-0. PubMed PMID: 34487721; PubMed Central PMCID: PMCPMC8443449.

23.	Sha R, Kong XM, Li XY, Wang YB. Global burden of breast cancer and attributable risk factors in 204 countries and territories, from 1990 to 2021: results from the Global Burden of Disease Study 2021. Biomark Res. 2024;12(1):87. Epub 2024/08/26. doi: 10.1186/s40364-024-00631-8. PubMed PMID: 39183342; PubMed Central PMCID: PMCPMC11346191.

24.	Global burden of 288 causes of death and life expectancy decomposition in 204 countries and territories and 811 subnational locations, 1990-2021: a systematic analysis for the Global Burden of Disease Study 2021. Lancet. 2024;403(10440):2100-32. Epub 2024/04/07. doi: 10.1016/s0140-6736(24)00367-2. PubMed PMID: 38582094; PubMed Central PMCID: PMCPMC11126520.

25.	Global burden and strength of evidence for 88 risk factors in 204 countries and 811 subnational locations, 1990-2021: a systematic analysis for the Global Burden of Disease Study 2021. Lancet. 2024;403(10440):2162-203. Epub 2024/05/19. doi: 10.1016/s0140-6736(24)00933-4. PubMed PMID: 38762324; PubMed Central PMCID: PMCPMC11120204.

26.	Global Burden of Disease Collaborative Network.Global Burden of Disease Study 2021 (GBD 2021) Socio-Demographic Index (SDI) 1950–2021. Seattle, United States of America: Institute for Health Metrics and Evaluation (IHME); 2024.

27.	Sha R, Kong XM, Li XY, Wang YB. Correction: Global burden of breast cancer and attributable risk factors in 204 countries and territories, from 1990 to 2021: results from the global burden of Disease Study 2021. Biomark Res. 2025;13(1):9. Epub 2025/01/12. doi: 10.1186/s40364-025-00729-7. PubMed PMID: 39799379; PubMed Central PMCID: PMCPMC11725199.
