# Supplementary material for: Global, regional, and national burden of hypertensive intracerebral hemorrhage, 1990 to 2021 and projections to 2050: Results from the Global Burden of Disease Study 2021
Source: PLoS One. 2025 Nov 18;20(11):e0336563. doi: 10.1371/journal.pone.0336563 (PMC12626279; doi:10.1371/journal.pone.0336563)
Supplement: Attachment — Submitted filename: Response to Reviewer.docx [file pone.0336563.s003.docx]

**Response to Reviewers**

**Manuscript ID number:**

PONE-D-25-19666

**Title of paper:**

Global, regional, and national burden of hypertensive intracerebral hemorrhage, 1990 to 2021 and projections to 2050: Results from the Global Burden of Disease Study 2021

Dear Editor and Reviewers,

We are again very grateful for the opportunity to revise the manuscript entitled “Global, regional, and national burden of hypertensive cerebral hemorrhage from 1990 to 2021 and projections to 2050: results from the Global Burden of Disease Study 2021” and would like to thank the reviewers again for their insightful comments. These comments were invaluable and very helpful in revising and refining the paper and provided important guidance for our research. In the submitted manuscript “Revised Manuscript with Track Changes”, we have retained all revision marks and provided detailed responses to the comments from the journal, reviewer 1 and reviewer 2, with the responses to reviewer 2 mainly marked in red. Below, we have provided a detailed response to each of the reviewers' comments and listed the number of lines of revision (for “Revised Manuscript with Track Changes” manuscripts) for ease of reference. In addition, we have completely revised the entire manuscript. In this response letter, reviewers' comments are italicized, and our corresponding revisions and additions to the manuscript are highlighted in red. We have endeavored to make all revisions clear and concise, and we hope that the revised manuscript will meet the requirements for publication.

***Journal Requirements:***

*Please review your reference list to ensure that it is complete and correct. If you have cited papers that have been retracted, please include the rationale for doing so in the manuscript text, or remove these references and replace them with relevant current references. Any changes to the reference list should be mentioned in the rebuttal letter that accompanies your revised manuscript. If you need to cite a retracted article, indicate the article’s retracted status in the References list and also include a citation and full reference for the retraction notice.*

**We have checked the reference list and this section is complete and correct and does not cite papers that have been retracted.**

**Response to reviewer #1:**

Dear Reviewer #1,

Thank you very much for your comments on the manuscript. Thank you for your time and effort in reviewing the revised version of my paper.

***Reviewer #1****: Good work. Authors have responded to reviewer comments appropriately. Global, regional, and national burden of hypertensive intracerebral hemorrhage, 1990 to 2021 and projections to 2050 is important to make changes in health policies considering the morbidity of hypertension in general population and its complications.*

**Once again, thank you very much for your comments on the revised manuscript. We have thoroughly proofread the manuscript to ensure that it meets the requirements for publication in your journal.**

**Response to reviewer #2:**

Dear Reviewer #2,

Thank you very much for your time and effort in providing valuable comments on our revised manuscript. We have revised the manuscript based on your feedback, and have elaborated on each of them below.

Thank you again for reviewing the manuscript, and I hope that my revisions will fulfill your requirements.

***Reviewer #2:*** *Thank you for submitting a revision and addressing the reviewer comments. I have reviewed your manuscript, and I think that this paper is very important and would benefit and add to the current literature.*

***Abstract:***

*In Lines 33–47, although the abstract is generally well-structured, these lines are slightly dense and long for an abstract; I suggest a clearer summarisation, for example, regional or country comparisons might be condensed and summarised as regional extremes in one concise sentence.*

**Thank you very much for your comments and suggestions. We have provided a clear and concise summary of the abstract, summarizing the extremes of the country and region in more concise sentences. In the “Revised Manuscript with Track Changes”, lines 47-57. The details of the modifications are as follows:**

**Country and regional patterns showed stark contrasts: Nauru and Mozambique had the highest ASDRs and age-standardized DALY rates, while Switzerland and Canada reported the lowest. Regionally, Central Africa, South Africa, Central Asia, East Asia, and Southeast Asia had the highest rates, whereas the Americas, Europe, and Oceania had the lowest. Age and gender trends indicated global peaks in the ASDRs (90–94 age group) and age-standardized DALY rates (85–89 age group), with men having higher rates across all age groups. Additionally, both ASDRs and age-standardized DALY rates were negatively associated with SDI levels. Projections from 2021 to 2050 suggest a continued overall decline in global ASDRs and age-standardized DALYs rates for HICH.**

***Introduction:***

*This is a very good and clear intor, however, it is subject for minor improvements. I found lines (78–91) very dense and lacking in flow and comprehensiveness. When I review papers, I usually suggest simplifying the meaning because if the paper is easy to read, it will be referenced a lot. Thus, I suggest that you summarise the key points and focus on their relevance to the HICH burden and disparities. Also, an important sentence to add is explaining why your paper is necessary despite previous GBD work. If you mention previous GBD work, what is different or important about your paper?*

**Thank you very much for your comments and suggestions. We have revised (78-91) to streamline the relevant exposition to make it more fluent, comprehensive, concise and easy to understand. In addition, we have added explanations of the necessity and importance of this thesis. In the “Revised Manuscript with Track Changes”, lines 101-110,123-132.** **The details of the modifications are as follows:**

**In 2021, high systolic blood pressure was a major contributor to high ASDRs and age-standardized DALYs. Other important risk factors include particulate matter pollution, smoking, indoor air pollution from solid fuels, and high sodium diets, and there are gender differences in these risks[1].Regionally, Central and Southeast Asia had a high prevalence of hypertension, exacerbated by unhealthy habits like high-salt diets and smoking, thus increasing the ICH burden[2]. In low SDI areas, household air pollution from solid fuels accounted for 40.2% of all ICH deaths, compared to only 8.1% and 5.9% for particulate matter pollution and smoking, respectively. In high SDI regions, deaths from these pollutants were lower[1].**

**Although most previous studies related to GBD [1] have focused on analyzing risk factors and distributional differences in ICH, this paper builds on previous studies by providing a detailed analysis of hypertension as an important risk factor and systematically analyzes the impact of geographic location, SDI, age, and sex differences on trends in ASDRs and age-standardized DALYs. To our knowledge, this GBD-based study is one of the most comprehensive studies to date analyzing the global burden of HICH in terms of mortality, DALYs, and projected future trends. These findings may inform public health interventions in specific regions, by gender, at different ages, etc., and may also provide valuable insights for the development of future prevention and management strategies.**

***Methods:***

*I think this section is very strong and defines how the paper has solid evidence. In line 121, insert a space after the URL. Otherwise, well done.*

**Again, thank you very much for your comment. We have inserted a space after the URL on line 121. In the “Revised Manuscript with Track Changes”, line 149.**

***Results:***

*This section is also well-written; however, I noticed that there were inconsistencies in terminology (for example, “countries” and “regions” are conflated); for instance, Southeast Asia is a region, not a country.*

**Again, thank you very much for commenting, we have double-checked this section for inconsistency and accuracy in wording. In the “Revised Manuscript with Track Changes”, lines 241-265.**

***Discussion:***

*This is a comprehensive and well-organised section. Still, there are a few areas that require refinement. Consider improving the transitions between paragraphs for a smoother flow, especially when shifting between global, regional, and national perspectives. In lines 343-344, referencing hormonal effects on neuroprotection is valuable; however, consider linking it to policy or clinical implications (e.g. gender-sensitive public health interventions). The final paragraph (lines 413-419) concludes well but could be enhanced by reiterating the practical importance of these findings, for example, how they might inform ongoing global health initiatives or SDG targets. The paragraph on limitations related to GBD data and methods (lines 427–447) is particularly strong; consider breaking it into two shorter paragraphs for improved readability. The Future Directions section is very strong; however, it would benefit from reordering for improved clarity. First outline research priorities, followed by the policy actions. For example, lines 457-461 (research) could precede lines 465–484 (policy).*

**Again, thank you very much for your comments. We have made the transition between global, regional and national perspectives more natural by adding transitional sentences and connecting words; we have linked hormonal effects on neuroprotection to public health interventions, In the “Revised Manuscript with Track Changes”, lines 370-376; and we have emphasized the importance of the study's findings for global health initiatives and the SDGs at the end to enhance the article's logic and usefulness, In the “Revised Manuscript with Track Changes”, lines 454-471.The details of the modifications are as follows:**

**Hormonal influences may explain some gender differences—estrogen’s neuroprotective effects in females delay ICH onset, underscoring the need for gender-sensitive interventions, such as tailored hypertension screening programs for men and women. Clinically, this could involve integrating sex-specific risk factor education into public health campaigns, particularly for smoking and air pollution exposure, which disproportionately affect males and females, respectively.**

**Our projections for the future indicate that the ASDR for HICH in 2050 will be 31.399 cases per 100,000 people, and the age-standardized DALYs rate will be 758.805 cases per 100,000 people. Although the global ASDR and age-standardized DALYs rate for HICH are on a downward trend, significant disparities persist across countries, regions, SDI levels, and genders. This prediction underscores the urgent need to prioritize targeted interventions in low-SDI and high-burden regions to align with Sustainable Development Goal 3 (SDG 3), which aims to reduce premature mortality from non-communicable diseases by one-third by 2030. For instance, the projected burden highlights the necessity of integrating HICH prevention into global health initiatives such as the World Health Organization’s Global Stroke Action Plan, particularly through initiatives like expanding hypertension screening in Central Sub-Saharan Africa and implementing gender-sensitive interventions to address male-female risk disparities. These findings also inform strategies for achieving universal health coverage (SDG 3.8), emphasizing the need to allocate resources toward healthcare infrastructure in regions with persistent high ASDRs, such as Southeast Asia and Oceania. By 2050, realizing these goals will require collaborative efforts to bridge care gaps, which our projections identify as critical for mitigating the ongoing global HICH burden.**

***Strengths and limitations:***

**Again, thank you very much for your comments. The paragraph on limitations of the GBD data and methodology (lines 427-447), we have split into two shorter paragraphs to improve readability. In the “Revised Manuscript with Track Changes”, lines 480-508.**

***Future directions***

**Again, thank you very much for your comments. We have reordered this section to make the exposition clearer. In the “Revised Manuscript with Track Changes”, lines 538-558. The details of the modifications are as follows:**

**Recommendations for future research and policy are essential to maintain and enhance HICH disease control. First, further research should focus on understanding the underlying causes of ASDR and age - standardized DALYs rate anomalies in regions like sub-Saharan Africa, examining socio-cultural, economic, and environmental factors, such as how local cultures, religious beliefs, and economic development influence HICH morbidity and mortality. Second, exploring the interactive effects of genetics, diet, and pollution on HICH risk across different SDI levels can provide a basis for formulating targeted prevention strategies. Third, evaluating the impact of emerging technologies, such as telemedicine and AI-based diagnostic tools, on HICH management in resource-constrained areas is crucial for optimizing care delivery.**

**Policy actions must build on these research directions to enhance HICH control. Low- and middle-SDI regions need to strengthen healthcare infrastructure, which includes integrating HICH screening into existing hypertension programs, training community health workers, and establishing telemedicine networks to serve rural areas. Policy initiatives should also prioritize formulating national HICH prevention plans and integrating them into public health strategies, expanding health insurance coverage for low-income groups and increasing the reimbursement for HICH-related medical expenses, promoting international cooperation to share prevention technologies and best practices, and implementing socio-economic development programs such as air pollution control, the provision of safe drinking water, and the construction of public fitness facilities to address upstream risk factors.**

Again, thank you very much for your comments on my paper. I appreciate your feedback and suggestions. I will revise my paper according to your comments and submit a revised version soon. I hope you will find the revised paper satisfactory and acceptable for publication. Thank you for your time and effort.

Sincerely, [Chao Zhang]

**Reference**

1.Song D, Xu D, Li M, Wang F, Feng M, Badr A, et al. Global, regional, and national burdens of intracerebral hemorrhage and its risk factors from 1990 to 2021. Eur J Neurol. 2025;32(1):e70031. Epub 2024/12/28. doi: 10.1111/ene.70031. PubMed PMID: 39731311; PubMed Central PMCID: PMCPMC11680743.

2.Schutte AE, Srinivasapura Venkateshmurthy N, Mohan S, Prabhakaran D. Hypertension in Low- and Middle-Income Countries. Circ Res. 2021;128(7):808-26. Epub 2021/04/02. doi: 10.1161/circresaha.120.318729. PubMed PMID: 33793340; PubMed Central PMCID: PMCPMC8091106.
