# Supplementary material for: Global, regional, and national burden of hypertensive intracerebral hemorrhage, 1990 to 2021 and projections to 2050: Results from the Global Burden of Disease Study 2021
Source: PLoS One. 2025 Nov 18;20(11):e0336563. doi: 10.1371/journal.pone.0336563 (PMC12626279; doi:10.1371/journal.pone.0336563)
Supplement: Attachment — Submitted filename: response to reviewer(1).docx [file pone.0336563.s004.docx]

**Response to Reviewers**

**Manuscript ID number:**

PONE-D-25-19666R2

**Title of paper:**

Global, regional, and national burden of hypertensive intracerebral hemorrhage, 1990 to 2021 and projections to 2050: Results from the Global Burden of Disease Study 2021

Dear Editor and Reviewers,

We are again very grateful for the opportunity to revise the manuscript entitled “Global, regional, and national burden of hypertensive cerebral hemorrhage from 1990 to 2021 and projections to 2050: results from the Global Burden of Disease Study 2021” and would like to thank the reviewers again for their insightful comments. These comments were invaluable and very helpful in revising and refining the paper and provided important guidance for our research. In the submitted manuscript “Revised Manuscript with Track Changes”, we have retained all revision marks and provided detailed responses to the comments from the journal, reviewer 1 and reviewer 2, with the responses to reviewer 2 mainly marked in red. Below, we have provided a detailed response to each of the reviewers' comments and listed the number of lines of revision (for “Revised Manuscript with Track Changes” manuscripts) for ease of reference. In addition, we have completely revised the entire manuscript. In this response letter, reviewers' comments are italicized, and our corresponding revisions and additions to the manuscript are highlighted in red. We have endeavored to make all revisions clear and concise, and we hope that the revised manuscript will meet the requirements for publication.

***Journal Requirements:***

*1.If the reviewer comments include a recommendation to cite specific previously published works, please review and evaluate these publications to determine whether they are relevant and should be cited. There is no requirement to cite these works unless the editor has indicated otherwise.*

**We have reviewed and evaluated the previously published works cited to ensure that they are relevant to what is being discussed.**

*2.Please review your reference list to ensure that it is complete and correct. If you have cited papers that have been retracted, please include the rationale for doing so in the manuscript text, or remove these references and replace them with relevant current references. Any changes to the reference list should be mentioned in the rebuttal letter that accompanies your revised manuscript. If you need to cite a retracted article, indicate the article’s retracted status in the References list and also include a citation and full reference for the retraction notice.*

**We have checked the reference list and this section is complete and correct and does not cite papers that have been retracted.**

**Response to reviewer #1:**

Dear Reviewer #1,

Thank you very much for your comments on the manuscript. Thank you for your time and effort in reviewing the revised version of my paper.

***Reviewer #1****: Good work. This article will help the literature on national burden of hypertensive intracerebral hemorrhage, 1990 to 2021 and projections to 2050.*

**Once again, thank you very much for your comments on the revised manuscript. We have thoroughly proofread the manuscript to ensure that it meets the requirements for publication in your journal.**

**Response to reviewer #2:**

Dear Reviewer #2,

Thank you very much for your time and effort in providing valuable comments on our revised manuscript. We have revised the manuscript based on your feedback, and have elaborated on each of them below.

Thank you again for reviewing the manuscript, and I hope that my revisions will fulfill your requirements.

***Reviewer #2:*** *Dear Author, thank you for choosing PLOS ONE and submitting your manuscript titled ‘Global, regional, and national burden of hypertensive intracerebral haemorrhage, 1990 to 2021 and projections to 2050: Results from the Global Burden of Disease Study 2021’. This is an important topic that adds to the current literature. Although I think this paper is good, I believe that there are minor improvements that could be addressed.*

***Introduction:***

*1.The introduction addresses the research question well and provides a good reference to the literature on global and local contexts. In lines 60-62 the sentence ‘.The sentence Hypertensive intracerebral haemorrhage (HICH) is the predominant… contains grammatical errors.*

**Thank you very much for your comments and suggestions. We have corrected lines 60-62 where it says "Hypertensive cerebral hemorrhage (HICH) is the main ...... " grammatical error. In the “Revised Manuscript with Track Changes”, lines 60-62. The details of the modifications are as follows:**

**Hypertensive intracerebral hemorrhage (HICH) is the predominant subtype of ICH, is strongly associated with hypertension，and accounts for approximately 70% of all ICH cases.**

*2.Although the information in lines Lines 64-73, althoug they is correct, the transition is somewhat confusing; consider writing ‘Despite improvements in clinical management, disparities remain...” to enhance the flow of the section.*

**We have revised the transition after lines 64-73 to “Despite improvements in clinical management, disparities remain ......” to enhance the flow of the section. In the “Revised Manuscript with Track Changes”, lines 77-84. The details of the modifications are as follows:**

**Despite improvements in clinical management, high systolic blood pressure, a major risk factor for HICH, continues to be strongly associated with factors such as smoking, diet, and the environment, and varies by country, region, gender, and SDI level^[1]^.**

*3.In reference 8, I think the figures provided require further clarification. I recommend specifying the populations studied or the source of these percentages for context because it is vague, and I am not sure how these figures are important or can be relied on.*

**We have further clarified the numbers provided in Reference 8 to be more specific about the populations studied or the source of the percentages, thus improving the credibility of the data. In the “Revised Manuscript with Track Changes”, lines 67-71. The details of the modifications are as follows:**

**Studies on the treatment of ICH have found that blood pressure control trials reduce ICH mortality by 10-15%^[2]^, after administration of anticoagulant-specific reversal agents (e.g., idarucizumab, prothrombin complex concentrate), mortality and hematoma enlargement rates in patients with ICH are reduced by approximately 30%-40% compared to baseline levels without reversal agents or conventional therapy^[3]^.**

*4**.**In lines 87-91, I noticed that the paragraph reiterates several ideas already mentioned earlier (e.g. high systolic blood pressure, regional variation, and SDI). I suggest condensing these points to avoid redundancy and to maintain reader engagement. also, when you mentioned “to bridge this gap,” I think it would be clearer to say exactly what gap they aim to address—such as “the lack of data on predictors of stroke length of stay in Indonesian hospitals.”*

**We have streamlined lines 87-91 where there is duplication from before. In addition, we further elaborated on what should be “to bridge this gap”. In the “Revised Manuscript with Track Changes”, lines 100-106. The details of the modifications are as follows:**

**Therefore, there is a need to explore why differences in spatial and temporal trends, gender, and age exist in HICH—for example, "in the Republic of Nauru, patients have low income levels and do not have access to standardized treatments^[4]^, which can complicate and make hospitalization unpredictable; in the Republic of Mozambique, there is a severe shortage of health workers^[5]^, which can prevent timely treatment and prolong hospitalization"—to guide prevention and treatment efforts.**

*5.Finally, I recommend refining the last sentence to be more specific and precise. Rather than stating that it will help “health service planning and management”, perhaps stating the improvement of discharge protocols or resource allocation would be better and more focused.*

**We have amended the last sentence to read “it could help to improve discharge protocols or resource allocation”. In the “Revised Manuscript with Track Changes”, lines 116-118. The details of the modifications are as follows:**

**These findings may inform public health interventions in specific regions, by gender, at different ages, etc., and can also help improve discharge protocols or resource allocation.**

***Methods:***

*1.This section is also well written and subject to minor suggestions. I suggest clarifying the data source in line 107 of the manuscript. It is not obvious how the February 2025 collection period applies when using retrospective GBD data. This could confuse the readers.*

**Again, thank you very much for your comment. We have elaborated on the data sources in line 107 and explained how the use of retrospective GBD data relates to the February 2025 collection period. In the “Revised Manuscript with Track Changes”, lines 121-125.The details of the modifications are as follows:**

**The data utilized in this study were derived from publicly available retrospective data from the GBD study. For the purpose of data extraction and collation in this manuscript, the specific operational period for accessing and organizing these GBD data was from February 5, 2025, to February 16, 2025.**

*2.In addition, I believe it is worth rephrasing line 114. Registering in a "paper proposal form" is vague. Do the authors mean the GBD disease proposal database? Clarify its purpose. The definition of HICH in the GBD dataset is not mentioned; therefore, I recommend briefly reminding readers how HICH was defined, even if it was described in prior publications.*

**The platform contacted on line 114 (https://uwhealthmetrics.co1.qualtrics.com/) is a questionnaire platform that is not directly linked to the GBD database and is the channel used to collate the data. Our data source is the Global Health Data Exchange query tool (GHDx) (http://ghdx.healthdata.org/gbd-results-tool). In addition, we describe the definition of HICH. In the “Revised Manuscript with Track Changes”, lines 133-137.The details of the modifications are as follows:**

**HICH is usually a spontaneous intracerebral hemorrhage that occurs in patients with a history of chronic hypertension (blood pressure ≥140/90 mmHg or a previous diagnosis of hypertension) and excludes cases caused by other etiologies (e.g., cerebral aneurysms, arteriovenous malformations, trauma, or coagulation disorders)** **^[6]^.**

*3.In lines 122-123, the reference to “prior publications” is too vague. We have named one major methodological reference and summarised the key methods used.*

**We have been more specific about “prior publications” in lines 122-123.In the “Revised Manuscript with Track Changes”, lines 143-152.The details of the modifications are as follows:**

**The data from the GBD2021 study were processed and analyzed as follows:Data were statistically analyzed and visualized using R 4.4.1 software. A linear regression model was used to calculate the EAPC and its 95% CI to assess the temporal trends of** **age-standardized rates (ASRs). A BAPC model was used to approximate marginal posterior distributions using the Integrated Nested Laplace Approximation (INLA) method to project ASDRs and age-standardized DALYs rates for HICH from 2025 to 2050^[7,8]^.**

*4.The information in lines 127–135 is repeated the same info twice. I suggest condensing to avoid redundancies. I felt confused reading this section because I am not sure why stratification is important in the context of HICH. I recommend providing a brief explanation.*

**We have removed and streamlined the repetitive information in lines 127-135 and explained the important role of SDI layering in HICH. In the “Revised Manuscript with Track Changes”, lines 168-171.The details of the modifications are as follows:**

**SDI stratification is critical to HICH research. It takes into account differences in health care access, prevention, and management across SDI levels and facilitates targeted analysis of socio-economic influences on HICH trends, thus helping to target interventions to specific regions.**

*5.In lines 154–156, the authors introduce limitations ("long-term predictions deviate…"), but I think this should be moved to the discussion or limitations section instead of being buried here.*

**We have deleted (“long-term predictions deviate...”) in lines 154-156 and moved it to the limitations section. In the “Revised Manuscript with Track Changes”, lines 518-521.**

***Results:***

*I think this section is comprehensive and data-rich. The figures and trends are clearly presented. In line 199, the phrase “a larger* *downward trend” is vague and could be supported by EAPC values to quantify the differences more clearly. Beside that, good job.*

**We apologize that we did not find the “larger downward trend” in the Results section after a careful search in and around line 199, which may be due to an oversight in our understanding of the comment. In order to improve the manuscript, I would like to ask you to point out the specific place where the change is needed, and I will immediately make the change according to your suggestion. I am deeply sorry for the inconvenience caused to you, and thank you again for your patience and guidance!**

***Discussion:***

*1.I think this is a well-structured and data-rich paper. I suggest rephrasing line 321 to emphasize the novelty more clearly for example, “This is the first study to explore HICH burden using updated 2021 GBD data stratified by SDI, age, and sex.” I suggest rephrasing line 321 to emphasize the novelty more clearly for example, “This is the first study to explore HICH burden using updated 2021 GBD data stratified by SDI, age, and sex.”*

**Again, thank you very much for your comments. We have revised the wording of line 321 to more clearly emphasize novelty. In the “Revised Manuscript with Track Changes”, lines 353-359. The details of the modifications are as follows:**

**This is the first study to explore HICH burden using updated 2021 GBD data stratified by SDI, age, and sex, and the findings have important value for the development of global health policy and the effective allocation of resources.**

*2.In lines 333-336, the explanation for sex-based trends is informative. However, I suggest clarifying that the differences in male and female trends may also reflect differences in healthcare access or behaviour, not just biology.*

**We have revised the interpretation of rows 333-336 based on gender trends to further clarify that differences in male and female trends may also reflect differences in health care access or behavior, not just biological differences. In the “Revised Manuscript with Track Changes”, lines 378-395. The details of the modifications are as follows:**

**In addition to biological factors, differences in gender trends are also reflected in access to health care and daily behaviors. Relevant studies have found that among hospital admissions, a higher proportion of men (85.6%) than women (74.7%) were admitted to acute stroke units or acute neurological intensive care units^[9]^, mostly due to men's busy schedules, low health awareness, and failure to seek medical attention in a timely manner, which resulted in more serious conditions. Smoking and excessive alcohol consumption are important risk factors for stroke, with men having more current smoking (23.3% vs. 4.2%) and excessive alcohol consumption (16.5% vs. 1.3%) behaviors than women, which in turn increases the risk of stroke^[10]^. This highlights the need for gender-sensitive interventions, such as tailoring hypertension screening programs for men and women. Clinically, this may require incorporating gender-specific risk factor education into public health campaigns, especially for men with low health awareness and smoking and excessive drinking behaviors.**

*3.In lines 349-351, I would suggest interpreting why Nauru and Mozambique show such high values as outliers due to data quality, small population size, or actual health system deficiencies.*

**We have provided an explanation of why Nauru and Mozambique show such high values in rows 349-351.In the “Revised Manuscript with Track Changes”, lines 403-409. The details of the modifications are as follows:**

**It is worth noting that the abnormally high values observed in Nauru and Mozambique may be due to limited data quality, small population size, prevalence of poor lifestyles^[11]^, weak health care systems and disease surveillance and defense systems^[12]^.**

*4.In line 403, I recommend adding 1-2 words on “how” oestrogen lowers lipid levels and affects vasodilation to strengthen biological plausibility.*

**We have added 2 words to line 403 to explain “how” estrogen lowers lipid levels and affects vasodilation to strengthen the biological plausibility. In the “Revised Manuscript with Track Changes”, lines 452-453. The details of the modifications are as follows:**

**mostly related to the neuroprotective effects of female gonadal hormones, which delay the onset of ICH in women by actively lowering blood lipid levels and directly altering the rapid vasorelaxation response of the blood vessel wall^[13]^.**

*5.In addition, in line 417, I recommend briefly stating what happened during the “reversal” in 1999–2003—was it due to war, policy changes, or data artefacts?*

**We have explained the reason for the 1999-2003 “reversal” in line 417. In the “Revised Manuscript with Track Changes”, lines 469-477. The details of the modifications are as follows:**

**This temporary reversal stems largely from the overlap of multiple factors. Large-scale geopolitical conflicts (e.g., the 1999 Kosovo War^[14]^, the 2003 Iraq War^[15]^, etc.) severely disrupted local health care systems-destroying infrastructure, creating supply shortages, and displacing populations-thereby raising regional disease burdens and skewing global aggregates. At the same time, transitional health policy reforms in some countries (e.g., China's experimental restructuring of health insurance^[16]^) led to temporary fluctuations in the accessibility of services and the effectiveness of disease management during this period. These combined factors drove short-term upward deviations in the long-term downward trajectory.**

*6.in lines 471-472, I think a concrete suggestion, such as using electronic health records or AI for surveillance, would enhance the future directions.*

**We have added specific recommendations to line 472 to strengthen future directions. In the “Revised Manuscript with Track Changes”, lines 531-540. The details of the modifications are as follows:**

**Research into the future should focus on assessing the impact of targeted health policies and equitable resource allocation while leveraging multidisciplinary approaches and advanced technologies—such as integrating electronic health records for real-time disease surveillance, applying AI in risk prediction models, developing AI-assisted diagnostic tools, and personalizing treatment recommendations through AI algorithms—to enhance HICH prevention, optimize clinical treatment options, and improve global health outcomes.**

Again, thank you very much for your comments on my paper. I appreciate your feedback and suggestions. I will revise my paper according to your comments and submit a revised version soon. I hope you will find the revised paper satisfactory and acceptable for publication. Thank you for your time and effort.

Sincerely, [Chao Zhang]

**Reference:**

1.	Xu L, Wang Z, Wu W, Li M, Li Q. Global, regional, and national burden of intracerebral hemorrhage and its attributable risk factors from 1990 to 2021: results from the 2021 Global Burden of Disease Study. BMC Public Health. 2024;24(1):2426. Epub 2024/09/07. doi: 10.1186/s12889-024-19923-7. PubMed PMID: 39243077; PubMed Central PMCID: PMCPMC11378620.

2.Wood H. Nature Reviews Neurology: the early years. Nat Rev Neurol. 2015;11(11):607. Epub 2015/11/05. doi: 10.1038/nrneurol.2015.204. PubMed PMID: 26530668.

3.Steiner T, Weitz JI, Veltkamp R. Anticoagulant-Associated Intracranial Hemorrhage in the Era of Reversal Agents. Stroke. 2017;48(5):1432-7. Epub 2017/04/13. doi: 10.1161/strokeaha.116.013343. PubMed PMID: 28400486.

4.	Carter K, Soakai TS, Taylor R, Gadabu I, Rao C, Thoma K, et al. Mortality trends and the epidemiological transition in Nauru. Asia Pac J Public Health. 2011;23(1):10-23. Epub 2010/12/21. doi: 10.1177/1010539510390673. PubMed PMID: 21169596.

5.	Feldacker C, Chicumbe S, Dgedge M, Augusto G, Cesar F, Robertson M, et al. Mid-level healthcare personnel training: an evaluation of the revised, nationally-standardized, pre-service curriculum for clinical officers in Mozambique. PLoS One. 2014;9(7):e102588. Epub 2014/07/30. doi: 1371/journal.pone.0102588. PubMed PMID: 25068590; PubMed Central PMCID: PMCPMC4113485.

6.Hemphill JC, 3rd, Greenberg SM, Anderson CS, Becker K, Bendok BR, Cushman M, et al. Guidelines for the Management of Spontaneous Intracerebral Hemorrhage: A Guideline for Healthcare Professionals From the American Heart Association/American Stroke Association. Stroke. 2015;46(7):2032-60. Epub 2015/05/30. doi: 10.1161/str.0000000000000069. PubMed PMID: 26022637.

7.	Global burden of 288 causes of death and life expectancy decomposition in 204 countries and territories and 811 subnational locations, 1990-2021: a systematic analysis for the Global Burden of Disease Study 2021. Lancet. 2024;403(10440):2100-32. Epub 2024/04/07. doi: 10.1016/s0140-6736(24)00367-2. PubMed PMID: 38582094; PubMed Central PMCID: PMCPMC11126520.

8.	Global burden and strength of evidence for 88 risk factors in 204 countries and 811 subnational locations, 1990-2021: a systematic analysis for the Global Burden of Disease Study 2021. Lancet. 2024;403(10440):2162-203. Epub 2024/05/19. doi: 10.1016/s0140-6736(24)00933-4. PubMed PMID: 38762324; PubMed Central PMCID: PMCPMC11120204.

9.	Roquer J, Rodríguez-Campello A, Jiménez-Conde J, Cuadrado-Godia E, Giralt-Steinhauer E, Vivanco Hidalgo RM, et al. Sex-related differences in primary intracerebral hemorrhage. Neurology. 2016;87(3):257-62. Epub 2016/06/10. doi: 10.1212/wnl.0000000000002792. PubMed PMID: 27281527; PubMed Central PMCID: PMCPMC4955279.

10.	Carcel C, Woodward M, Wang X, Bushnell C, Sandset EC. Sex matters in stroke: A review of recent evidence on the differences between women and men. Front Neuroendocrinol. 2020;59:100870. Epub 2020/09/04. doi: 10.1016/j.yfrne.2020.100870. PubMed PMID: 32882229.

11.	Schooneveldt M, Songer T, Zimmet P, Thoma K. Changing mortality patterns in Nauruans: an example of epidemiological transition. J Epidemiol Community Health. 1988;42(1):89-95. Epub 1988/03/01. doi: 10.1136/jech.42.1.89. PubMed PMID: 3418293; PubMed Central PMCID: PMCPMC1052687.

12.	Schwitters A, Lederer P, Zilversmit L, Gudo PS, Ramiro I, Cumba L, et al. Barriers to health care in rural Mozambique: a rapid ethnographic assessment of planned mobile health clinics for ART. Glob Health Sci Pract. 2015;3(1):109-16. Epub 2015/03/07. doi: 10.9745/ghsp-d-14-00145. PubMed PMID: 25745124; PubMed Central PMCID: PMCPMC4356279.

13. Roof RL, Hall ED. Gender differences in acute CNS trauma and stroke: neuroprotective effects of estrogen and progesterone. J Neurotrauma. 2000;17(5):367-88. Epub 2000/06/01. doi: 10.1089/neu.2000.17.367. PubMed PMID: 10833057.

14.	Ashford MW, Gottstein U. The impact on civilians of the bombing of Kosovo and Serbia. Med Confl Surviv. 2000;16(3):267-80. Epub 2000/10/19. doi: 10.1080/13623690008409524. PubMed PMID: 11037273.

15.	Lafta R, Al-Shatari S, Cherewick M, Galway L, Mock C, Hagopian A, et al. Injuries, Death, and Disability Associated with 11 Years of Conflict in Baghdad, Iraq: A Randomized Household Cluster Survey. PLoS One. 2015;10(8):e0131834. Epub 2015/08/08. doi: 10.1371/journal.pone.0131834. PubMed PMID: 26252879; PubMed Central PMCID: PMCPMC4529175.

16.	Huang F, Gan L. The Impacts of China's Urban Employee Basic Medical Insurance on Healthcare Expenditures and Health Outcomes. Health Econ. 2017;26(2):149-63. Epub 2015/11/04. doi: 10.1002/hec.3281. PubMed PMID: 26524988.
